# Supplementary material for: Paradoxical dominant negative activity of an immunodeficiency-associated activating PIK3R1 variant
Source: bioRxiv. 2023 Nov 2:2023.11.02.565250. Preprint. [Version 1] doi: 10.1101/2023.11.02.565250 (PMC10705566; doi:10.1101/2023.11.02.565250)
Supplement: Supplementary file 1 [file media-1.docx]

### Appendix

**Paradoxical dominant negative activity of an immunodeficiency-associated activating** ***PIK3R1*** **variant**

Tomlinson *et al*

### Contents

	### Page

### Expanded View Figure EV1……………………………………………………..……..	2 

### Expanded View Figure EV2……………………………………………………..……..	3 

### Expanded View Figure EV3……………………………………………………..……..	4

### Expanded View Figure EV4……………………………………………………..……..	5

### Expanded View Figure EV5……………………………………………………..……..	6 

### Expanded View Figure EV6……………………………………………………..……..	7-8

### Expanded View Figure EV7……………………………………………………..……..	9-10

### Expanded View Figure EV8……………………………………………………..……..	11-12

### Expanded View Table EV1………………………………………..……………..……..  13

Expanded Materials and Methods**………………………………………………………**	14

### Figure EV1. Further characterisation of primary dermal fibroblasts studied. (A) Details of cDNA sequence for *PIK3CA* and *PIK3R1* from cells derived from healthy controls (WT), patients with APDS2 (p85α ΔEx11) or *PIK3CA*-related overgrowth syndrome (PROS), confirming expected expression of mutant alleles. (B) Higher magnification detail of immunoblot from wild-type and APDS2 fibroblasts showing truncated p85α Δex11. (C) All 3 immunoblot replicates for p110δ blots quantified in Figure 1, showing severely reduced p110δ expression in the APDS2 cell line.

###  

###      

### Figure EV2. Full statistical analysis of data presented in main Figure 1 Analysis of insulin-induced increase in (A) AKT S473/4 and (B) T308/9 phosphorylation. The paired mean difference for 3-4 comparisons are shown in Cumming estimation plots. The raw data, as presented in Figure 1, are re-plotted on the upper axes with paired observations connected by a line. On the lower axes, paired mean differences are plotted as a bootstrap sampling distribution. Mean differences are depicted as dots; 95% confidence intervals are indicated by the ends of the vertical error bars. (C-D) Analysis of differences in (C) p110α and (D) p110δ protein expression between healthy control cells and cells from PROS patients harbouring activating PIK3CA mutations. Mean differences are shown in Gardner-Altman estimation plots, with expression data plotted on the left axes and mean difference on floating axes on the right, again as a bootstrap sampling distribution with mean difference depicted as a dot and 95% confidence intervals by the ends of the vertical bar.

###   

###  

### Figure EV3: Full statistical analysis of data presented in main Figure 2.  Analysis of the effects of doxycycline-induced expression of wild-type (WT) or ΔEx11 (APDS2) p85α, or of p110α H1047R (PROS) on Akt S473/4 (A,B)  and T308/9 (C,D) phosphorylation. Comparisons are made in both the basal, non-insulin stimulated state (A,C) and after stimulation with 10 nmol/L insulin (B,D). Paired mean differences for 3 comparisons are shown in Cumming estimation plots. Raw data, as presented in Figure 2, are re-plotted on the upper axes with paired observations connected by a line. On the lower axes, paired mean differences are plotted as a bootstrap sampling distribution. Mean differences are depicted as dots; 95% confidence intervals are indicated by the ends of the vertical error bars.

### Figure EV4. The effect of graded expression of wild-type or disease-associated p85α on 3T3-L1 preadipocytes. Immunoblots of p85α, phosphoAkt (S473) and total Akt are shown for control 3T3-L1 cells and 3T3-L1 cells conditionally expressing wild-type (WT), or APDS2-associated mutant p85α under the control of doxycycline (Dox), with and without 10 minutes of exposure to insulin as indicated. The filled black triangles indicate increasing concentrations of doxycycline (from left to right: 0, 0.02, 0.03, 0.045, 0.065, or 0.1 μg/mL). Exposure was for 72 hours in all cases. The truncated p85α variant can be seen below the WT p85α for the APDS2 ΔEx11 mutant.

###  

### Figure EV5. Full statistical analysis of data presented in main Figure 3. Analysis of fluorescence polarisation assay of phosphoinositide 3-kinase (PI3K) activity of *in vitro* synthesised wild-type (WT) or mutant (E489K, R649W or Y657X) p85α. Results for p110α- and p110β-containing PI3K are shown in (A) and (B) respectively. All data were acquired in the presence of phosphotyrosine peptide. Paired mean differences for 3 comparisons are shown in Cumming estimation plots. Raw data, as presented in Figure 3, are re-plotted on the upper axes with paired observations connected by a line. On the lower axes, paired mean differences are plotted as a bootstrap sampling distribution. Mean differences are depicted as dots; 95% confidence intervals are indicated by the ends of the vertical error bars. Results for the R649W p85α mutation only are shown with p110δ in (C). In this case raw data are re-plotted on the left hand axes with paired observations connected by 3 nearly superimposed lines. On the right hand axes, paired mean differences are plotted as a bootstrap sampling distribution.

### Figure EV6. Full statistical analysis of data presented in main Figure 5. Analysis of the effects of doxycycline (dox)-induced expression of wild-type (WT), Y657X, R649W or ΔEx11 p85α on association of p110α (A,B)  and p85α (C,D) with Irs1. Results of co-immunoprecipitation with (B,D) and without exposure to 10 nmol/L insulin Comparisons are made in both the basal, non-insulin stimulated state (A,C) and after stimulation with 10 nmol/L insulin (B,D) are shown. Paired mean differences for 3 comparisons are shown in Cumming estimation plots. Raw data, as presented in Figure 5, are re-plotted on the upper axes with paired observations connected by a line. On the lower axes, paired mean differences are plotted as a bootstrap sampling distribution. Mean differences are depicted as dots; 95% confidence intervals are indicated by the ends of the vertical error bars.

 

**Figure EV7. Attenuated insulin-induced association of p110****α** **with Irs2 in the presence of APDS2 and SHORT syndrome mutant PIK3R1.** Results of immunoblotting of anti-Irs2 immunoprecipitates from 3T3-L1 cells expressing wild-type, APDS2-associated or SHORT syndrome-associated mutant p85α under the control of doxycycline (Dox) are shown. Treatment with 100nM insulin (Ins) is indicated. (A) One representative immunoblot of immunoprecipitate, cell lysate prior to immunoprecipitation, and post immunoprecipitation supernatant is shown. Two separate sets of gels, including independent wild-type controls are shown on left and right. (B-C) Quantification of immunoblot bands from immunoprecipitates from 3 independent experiments. Immunoprecipitated p110α is shown normalized to immunoprecipitated Irs2 from all 3 independent experiments in (B), and immunoprecipitated p85α similarly in (C). Datapoints from the same experiment +/- insulin are connected by lines. Asterisks indicate significant differences induced by transgene overexpression (i.e. plus versus minus doxycycline). 95% confidence intervals for the paired mean differences for these comparisons are shown in Figure EV8.

### Figure EV8. Full statistical analysis of data presented in Figure EV7. Analysis of the effects of doxycycline (dox)-induced expression of wild-type (WT), Y657X, R649W or ΔEx11 p85α on association of p110α (A,B)  and p85α (C,D) with Irs2. Results of co-immunoprecipitation with (B,D) and without exposure to 10 nmol/L insulin Comparisons are made in both the basal, non-insulin stimulated state (A,C) and after stimulation with 10 nmol/L insulin (B,D). Paired mean differences for 3 comparisons are shown in Cumming estimation plots. Raw data, as presented in Figure EV7, are re-plotted on the upper axes with paired observations connected by a line. On the lower axes, paired mean differences are plotted as a bootstrap sampling distribution. Mean differences are depicted as dots; 95% confidence intervals are indicated by the ends of the vertical error bars. 

| **Antibody** | **Company** | **Catalogue No.** | **Species** | **Dilution used (immunoblot/IP)** |
| --- | --- | --- | --- | --- |
| p85α | CST | 4257 | Rabbit | 1:1000 |
| Phospho-AKT/Akt (T308) | CST | 9275 | Rabbit | 1:1000 |
| Phospho-AKT/Akt (S473) | CST | 9271 | Rabbit | 1:1000 |
| AKT/Akt (1,2,3) | CST | 2920 | Mouse | 1:1000 |
| p110α | CST | 4249 | Rabbit | 1:1000/1:50 |
| p110δ | CST | 34050 | Rabbit | 1:1000 |
| Irs1 | Millipore | 06-248 | Rabbit | 1:500 |
| Irs1 | CST | 2382 | Rabbit | 1:1000/1:50 |
| Irs2 clone 9.52 | Millipore | MABS15 | Mouse | 1:1000/1:50 |
| β-actin | CST | 4967 | Rabbit | 1:5000 |
| Anti-rabbit IgG; HRP-linked | CST | 7074 | Goat | 1:5000 |
| Anti-mouse IgG; HRP-linked | CST | 7076 | Horse | 1:5000 |

 **Table EV1: Antibodies Used**

**Expanded Materials and Methods**

*Baculovirus Generation*

p85α point mutation expression constructs were created by site-directed mutagenesis of a pACEBac1_Homo sapiens p85α plasmid using a QuikChange II XL Site-Directed Mutagenesis Kit. In-Fusion cloning was used to generate pACEBac1_p85α ΔEx11. In brief, pACEBac1_p85α backbone was digested (BamHI/NotI) and purified using Zymoclean Gel DNA Recovery Kit, while cDNA insert was purified using NucleoSpin® Clean-up Kit (Takara). Insert generation was performed by High-Fidelity PCR using primers designed to yield fragments containing exons 1-10 or 12-16 with the necessary overlap, with inserts verified by electrophoresis and purified using NucleoSpin® Clean-up kit. In-Fusion reactions were performed according to manufacturer’s guideline (Takara) using 100ng each of linearised plasmid and both inserts in 10μL. Products were transformed into Stellar Competent cells (Takara). Purified plasmid inserts were sequenced and verified by restriction enzyme digest, exploiting loss of a DpnI site within excised exon 11.

pACEBac1_p85α and pFastBac™HT B plasmids encoding N terminally tandem His-tagged p110 subunits were used to generate bacmid DNA. MAX Efficiency DH10Bac™ Competent or EMBacY cells (Thermofisher) were transformed with 40ng plasmid DNA and turbid cultures plated onto agar containing 50μg/mL kanamycin, 10μg/mL gentamicin, 10μg/mL tetracycline, 100μg/mL X-Gal and 40μg/mL IPTG. Single colonies were picked, expanded and purified at 2 days and bacmid concentrations quantified by Nanodrop 1000 Spectrophotometer (Thermo Scientific). 2-4μg bacmid was transfected into Sf9 cells in 6 well plates using Insect-XPRESS (Promega E2311). Cells were incubated at 2°C for 5 days and bacmid YFP expression assessed by Leica DM IL LED Fluo microscope using a green fluorescent protein filter cube. Pooled virus from 2 wells of supernatant (P1 stock) was used to produce high titre P2 stock by transfection of 1.5x10^6^ Sf9 cells/mL (Thermofisher) in 450mL using Insect-XPRESS with L-Glutamine (Lonza LZBE12-730Q). For catalytic subunits, 1-2mL P2 stock was used for a further round of Sf9 transfection and expansion to generate P3 stock.

*Purification of PI3K holoenzymes*

1.5-2L of 1.5x10^6^ Sf9 cells/mL were co-infected with 18mL P3 catalytic subunit and 4mL P2 regulatory subunit baculovirus. Non infected Sf9 cells were negative controls, and a prior protein preparation of 320 kDa was the positive control. Cells were cultured at 27°C, harvested 48h post-infection, pelleted, washed and stored at -80°C. Pellets were later lysed by sonication in buffer containing 20 mM TrisHCl (pH 8.0), 300 mM NaCl, 20 mM imidazole (pH 8.0) and 1 mM TCEP (pH 7.0) at 4 °C. Universal Nuclease (Thermofisher) was added to lysates before ultracentrifugation at 35,000 rpm for 35min at 4°C. p85α/p110 heterodimers were pulled down via 6 tandem p110 N-terminal His tags, preventing purification of monomeric p85α, using tandem Ni^2+^HisTrap Fast Flow columns (GE Healthcare) (equilibration buffer 20 mM TrisHCl (pH 8.0), 100 mM NaCl, 20 mM imidazole and 1 mM TCEP (pH 7.0); elution buffer 20 mM TrisHCl (pH 8.0), 100 mM NaCl, 200 mM imidazole and 1 mM TCEP (pH 7.0)). Further purification utilised a heparin HiTrap Q HP column (GE Healthcare) equilibrated with 20mM TrisHCl (pH 8.0) and 1mM TCEP (pH 7.0), with proteins eluted in 20mM TrisHCl (pH 8.0), 1 M NaCl and 1mM TCEP (pH 7.0). Eluted fractions were concentrated to ≥1mg/mL using Millipore Amicon Ultra-15 Centrifugal Filter Units with Ultracel-50 membrane, and 1mL concentrated fractions were gel filtered on Superdex 200 16/60 columns (GE Healthcare) equilibrated in 20mM HEPES (pH 7.5), 100mM NaCl and 2mM TCEP (pH 7.0). The p110 6-His tag was retained for proteins used in functional analyses. KTA Protein Purification Systems (GE Healthcare) and UNICORN Control Software version 5.11 (GE Healthcare) were used for all purifications. Purity of eluted complexes was verified by SDS-PAGE, and purified proteins were quantified using a Nanodrop 1000 Spectrophotometer (Thermofisher). Protein concentration was calculated using the molecular extinction coefficient (assuming full cysteine reduction) determined by heterodimer sequence input to the ProtParam tool (ExPASy). Purified proteins were stored at –80°C in single-use aliquots.

Total injection volume onto gel filtration columns was kept at 1 mL. Yields of PI3K complexes were determined using the area under the curve (280nm mAU per mL eluted protein) from gel filtration chromatograms, and normalised to the volume of Sf9 cells infected.

*Liposome preparation*

Chloroform/methanol solutions of sphingomyelin, cholesterol, porcine brain phosphatidylcholine, porcine brain phosphatidylethanolamine, porcine brain phosphatidylserine and porcine brain PI(4,5)P_2_ were mixed to generate a preparation containing 5/10/15/45/20/5% of each lipid, with a total lipid concentration of 5 mg/mL and final PI(4,5)P_2_ concentration 250μg/mL. Lipid preparations were dessicated under argon and then in a vacuum desiccator. Lipids were rehydrated in buffer containing 20mM HEPES (pH 7.5), 100 mM KCl and 1 mM EGTA (pH 8.0) using vortexing for 3 min, waterbath sonication for 15 min, and 10 cycles between liquid nitrogen and a 43°C water bath. Unilamellar vesicles were generated by extrusion through polycarbonate filters with 100nm pores, using a glass-tight syringe. Single use aliquots were stored at -80 °C.

*Fluorescence Polarisation Assay*

PI(3,4,5)P_3_ production was measured by modified PI3-Kinase activity fluorescence polarisation assay (Echelon Biosciences, Salt Lake City, UT, USA). 10μL reactions in 384-well black microtitre plates used 1mM liposomes containing 50μM PI(4,5)P_2_, optimised concentrations of purified PI3K proteins, 100μM ATP, 2mM MgCl_2_, with or without 1μM tyrosine bisphosphorylated peptide derived from PDGFRβ (“pY2”; Cambridge peptides). Reactions were quenched with 5μL of solution containing 20mM HEPES (pH 7.5), 150mM NaCl, 30mM EDTA (pH 7.4) and 400nM GST–Grp1-PH, followed by addition of 5μL 40nM TAMRA Red Fluorescent Probe in HNT buffer. Identity of the lipid group coupled to the TAMRA probe was not disclosed by Echelon Biosciences, but as Grp1 recognises specific lipid head groups in competition with PI(3,4,5)P_3_, it is likely to be a lipid with a similar head group such as inositol 1,3,4,5-tetrakisphosphate. After 1h equilibration, fluorescence polarisation was measured using a PHERAstar spectrofluorometer (BMG Labtech, Ortenberg, Germany) with the FP/540-20/590-20/590-20 optical module. The concentrations of wild-type protein complexes used in the assay were: 20-40nM p85α/p110α, 80-160nM p85α/p110β and 80-160nM p85α/p110δ for basal activities, and 1-2nM p85α/p110α, 2-4 nM p85α/p110β and 2-6nM p85α/p110δ for pY2-stimulated activities. Concentrations of thawed protein aliquots were assessed as 280 nm absorbance by Nanodrop 1000 Spectrophotometer, and samples of preparations used in assays were resolved by SDS-PAGE to confirm equal amounts of each complex. Three technical replicates were used per assay on at least three different occasions. Relevant wild-type p85α/p110 catalytic isoforms were included in each assay as controls. Other controls were 1. Stop mix + probe without lipids, to assess maximum fluorescence polarisation. 2. Stop mix + probe with lipids, to determine the effect of lipids on maximum fluorescence polarisation. 3. Probe without stop mix or lipids to determine minimum fluorescence polarisation and 4. Probe with lipids but no stop mix to determine the effect of lipids on minimum fluorescence polarisation. Standard curves of diC8-PI(3,4,5)P_3_ in the presence of liposomes were included in every experiment and used to infer PIP_3_ generated by purified PI3K. Graphpad Prism v6.0 was used to generate sigmoidal standard curves by plotting log transformed diC8-PI(3,4,5)P3 concentrations against fluorescence polarisation. Standard curves for each experiment provided the linear range within which PIP_3_ could be accurately determined. Preliminary studies found only trivial differences in measured fluorescence polarisation in the presence of pY2, which was thus omitted from subsequent standard curves. Quadruplicate interpolated PIP_3_ concentrations were averaged and normalised by reaction time (30 min) and enzyme concentration in pM to yield PI3K activity in pmol PIP_3_/min/pM enzyme. Specific activities were then further normalised to activity of p85α wild-type controls in the presence of pY2 in each experiment.

*Insulin signalling studies*

3T3-L1 cells serum starved in DMEM containing 0.5 % BSA for 16h were stimulated with 100nM Actrapid insulin (Novo Nordisk) for 10min. Monolayers were snap frozen in liquid N_2_ and stored at -80°C. 200-600μL co-IP lysis (20mM HEPES, 150mM NaCl, 1.5mM MgCl_2_, 10% (v/v) glycerol, 1% (v/v) Triton X-100, 1mM EGTA pH 7.4, 1mM PMSF, 2mM activated sodium orthovanadate, Complete Mini EDTA-free protease inhibitor cocktail and PhosSTOP, in Milli-Q Ultrapure water (Millipore)) or RIPA buffer (50mM Tris HCl pH 8.0, 150mM NaCl, 1% NP-40, 0.5% sodium deoxycholate, 0.1% SDS with added Complete Mini EDTA-free protease inhibitor cocktail and PhosSTOP, in Milli-Q Ultrapure Water (Millipore)) was added to frozen cells before scraping of lysate into pre-chilled tubes, incubation on ice for 30min and clearing by centrifugation. Protein was quantified using the Bio-Rad DC assay. 

Lysates were mixed with NuPAGE® SDS Loading buffer supplemented with 5% β-mercaptoethanol and boiled before SDS-PAGE. For co-immunoprecipitation (Co-IP) lysates (150-300μg protein) were incubated in 500μL Co-IP buffer with immunoprecipitating antibody overnight at 4°C. 10μg lysate mixed with NuPAGE® SDS Loading buffer and 5% β-mercaptoethanol was stored to represent Co-IP input. Co-IP samples were incubated for 2h with 1.5mg PBST-washed Protein G Dynabeads® (Life Technologies) at 4°C before centrifugation and bead removal using a DynaMag™-2 Magnet (Invitrogen). Supernatants mixed 1:1 with Co-IP elution buffer (2X NuPAGE® LDS, 100 mM NuPAGE® Sample Reducing Agent, in Co-IP lysis buffer) were boiled for 10 min. Beads were washed 5 times in 50μL Co-IP lysis buffer, before protein elution with 25μL Co-IP Elution buffer and boiling for 10min. Input, supernatant and co-IP samples underwent SDS-PAGE using NuPAGE® 4-12% gradient Bis-Tris gels in NuPAGE® MOPS SDS Running Buffer. Transfer to nitrocellulose was performed using the iBlot™ Dry Blotting System (Invitrogen), with preincubation in Equilibration Transfer Buffer for proteins above 150kDa. 

For immunoblotting, blocked membranes were incubated overnight at 4°C in primary antibody, washed and incubated with HRP-linked anti-rabbit or anti-mouse IgG secondary antibody diluted 1:5000 in blocking buffer. Proteins were visualized using the ChemiDoc™ MP System (Bio-Rad) and band intensities quantified using Image Lab software (Bio-Rad).
